# Intravascular imaging in peripheral arterial disease: a contemporary literature review

**DOI:** 10.1093/ehjopen/oeag016

**Published:** 2026-02-13

**Authors:** Jason Galo, Abdullah Al-Qaraghuli, Ryan Wallace, Aninka Saboe, Julianna Morera, Pablo Rubio, Abhishek Chaturvedi, Beni Verma, Kalyan R Chitturi, Ron Waksman, Hector M Garcia-Garcia

**Affiliations:** Section of Interventional Cardiology, MedStar Washington Hospital Center, 110 Irving street, NW, Washington, DC 20010, USA; Department of Internal Medicine, MedStar Washington Hospital Center, 110 Irving street, NW, Washington, DC 20010, USA; Department of Cardiology, MedStar Washington Hospital Center, 110 Irving street, NW, Washington, DC 20010, USA; Section of Interventional Cardiology, MedStar Washington Hospital Center, 110 Irving street, NW, Washington, DC 20010, USA; College of Arts and Sciences, University of Miami, 1252 Memorial Dr, Coral Gables, Miami, FL 33146, USA; Section of Interventional Cardiology, MedStar Washington Hospital Center, 110 Irving street, NW, Washington, DC 20010, USA; Section of Interventional Cardiology, MedStar Washington Hospital Center, 110 Irving street, NW, Washington, DC 20010, USA; Section of Interventional Cardiology, MedStar Washington Hospital Center, 110 Irving street, NW, Washington, DC 20010, USA; Section of Interventional Cardiology, MedStar Washington Hospital Center, 110 Irving street, NW, Washington, DC 20010, USA; Section of Interventional Cardiology, MedStar Washington Hospital Center, 110 Irving street, NW, Washington, DC 20010, USA; Section of Interventional Cardiology, MedStar Washington Hospital Center, 110 Irving street, NW, Washington, DC 20010, USA

**Keywords:** Intravascular ultrasound, Optical coherence tomography, Peripheral arterial disease, Peripheral endovascular intervention, Balloon angioplasty, Stenting

## Abstract

Intravascular ultrasound and optical coherence tomography are advanced intravascular imaging modalities that provide detailed vessel assessment and have shown potential to optimize peripheral arterial disease interventions. While angiography remains the standard guidance tool during peripheral endovascular procedures, intravascular imaging offers superior visualization of lesion characteristics and vessel dimensions. This scoping review of the literature, conducted using MEDLINE and EMBASE from inception to 21 September 2025 and reported according to PRISMA-ScR recommendations, evaluates the technology, principles, and clinical applications of intravascular ultrasound (IVUS) and optical coherence tomography (OCT) in peripheral interventions. Compared with angiography alone, both modalities aid in lesion preparation, improve stent implantation, reduce procedural complications, and are associated with improved vessel patency and lower reintervention rates. Optical coherence tomography, owing to its higher resolution, is particularly useful for detailed lesion morphology assessment and identification of neointimal hyperplasia in in-stent restenosis, although its use is limited by reduced penetration depth, field of view, contrast requirements, and the need for blood clearance. Overall, IVUS and OCT facilitate precise vessel characterization and improved procedural outcomes in peripheral arterial disease, though further high-quality randomized studies are needed to define optimal indications and cost-effectiveness.

## Introduction

Lower extremity peripheral arterial disease (PAD) can manifest in various ways, including lower extremity claudication and functional impairment, frequently leading to its most severe form, limb amputation.^1,2^ Many patients will require peripheral endovascular intervention (PEI) for persistent, limiting claudication or to prevent limb amputation in cases of chronic limb-threatening ischaemia (CLTI) or acute limb ischaemia (ALI).^1,3,4^ Revascularization is consistently associated with lower major amputation rates and better amputation free survival,^3,5,6^ while effects on mortality are mixed across studies and not proven in randomized controlled trials (RCTs). Current guidelines recommend revascularization for patients with lifestyle limiting claudication despite maximal medical therapy, for patients with CLTI to minimize tissue loss, and for patients with ALI for limb salvage.^7^

PAD is characterized by a high prevalence of fibrocalcific plaques, medial calcification, and small artery calcification.^8^ Luminal thrombus in PAD often occurs independently of underlying atherosclerotic disease, in contrast to coronary artery disease (CAD), where it is typically a direct consequence of plaque disruption.^9^ Femoropopliteal lesions are more commonly associated with acute thrombi and atherosclerotic plaques, whereas infra-popliteal lesions demonstrate more chronic thrombi and a greater degree of medial calcification.^8,10^

Plaque morphology assessment is essential for procedural planning.^11,12^ Intravascular imaging modalities such as intravascular ultrasound (IVUS) and optical coherence tomography (OCT) enable detailed characterization of plaque composition and structure, including soft plaque, intimal and medial calcification, thrombus, lesion length, chronic total occlusions, and in-stent restenosis.^11,12^ Imaging modalities provide superior intraluminal visualization compared to angiography, allowing for more accurate vessel sizing, lesion length measurement, and detection of complications, which directly informs device selection and procedural strategy^11,12^

Calcified and long lesions are particularly challenging, as they are associated with higher rates of restenosis and procedural failure, especially with drug-coated balloons (DCB), which require adequate vessel preparation for optimal drug delivery.^12,13^ Vessel preparation failure (e.g. residual stenosis, severe dissection) is a key predictor of poor outcomes with DCBs, while high plaque burden and popliteal location are risk factors for restenosis with drug-eluding stents (DES).^13^

The choice between DCB and DES is influenced by plaque morphology. Both devices show superior patency and lower target lesion revascularization (TLR) compared to plain balloon angioplasty or bare-metal stents, and their relative efficacy is similar in complex lesions, with DCBs often requiring adjunctive atherectomy or bailout stenting^14–16^. The need for adjunctive procedures is higher with DCBs in heavily calcified or long lesions.^15^

Angiography, while standard, is limited by its two-dimensional nature and inability to accurately assess vessel diameter, plaque burden, calcification, and dissections. It often underestimates lesion length and severity, leading to suboptimal device sizing and placement.^17,18^ The purpose of this review is to discuss the totality of current literature regarding the use of intravascular imaging (IVUS and OCT) in PEI.

## Methods

A scoping review of the literature was performed using MEDLINE and EMBASE from inception through 21 September 2025. The review was designed to identify, categorize, and summarize existing evidence on the use of intravascular imaging in peripheral endovascular interventions across study designs, clinical indications, and reported outcomes. The conduct and reporting of this review followed established guidance for scoping reviews and adhered to PRISMA-ScR recommendations.^19^

The search strategy included controlled vocabulary and free-text terms related to intravascular imaging and peripheral arterial disease. Keywords included ‘intravascular ultrasound (IVUS)’ and ‘optical coherence tomography (OCT)’ in combination with ‘peripheral arterial disease (PAD),’ ‘endovascular interventions,’ ‘chronic limb-threatening ischemia,’ ‘claudication,’ ‘balloon angioplasty,’ ‘stenting,’ and ‘outcomes.’ Reference lists of relevant reviews and included studies were manually screened to identify additional eligible articles.

**Figure 1:**
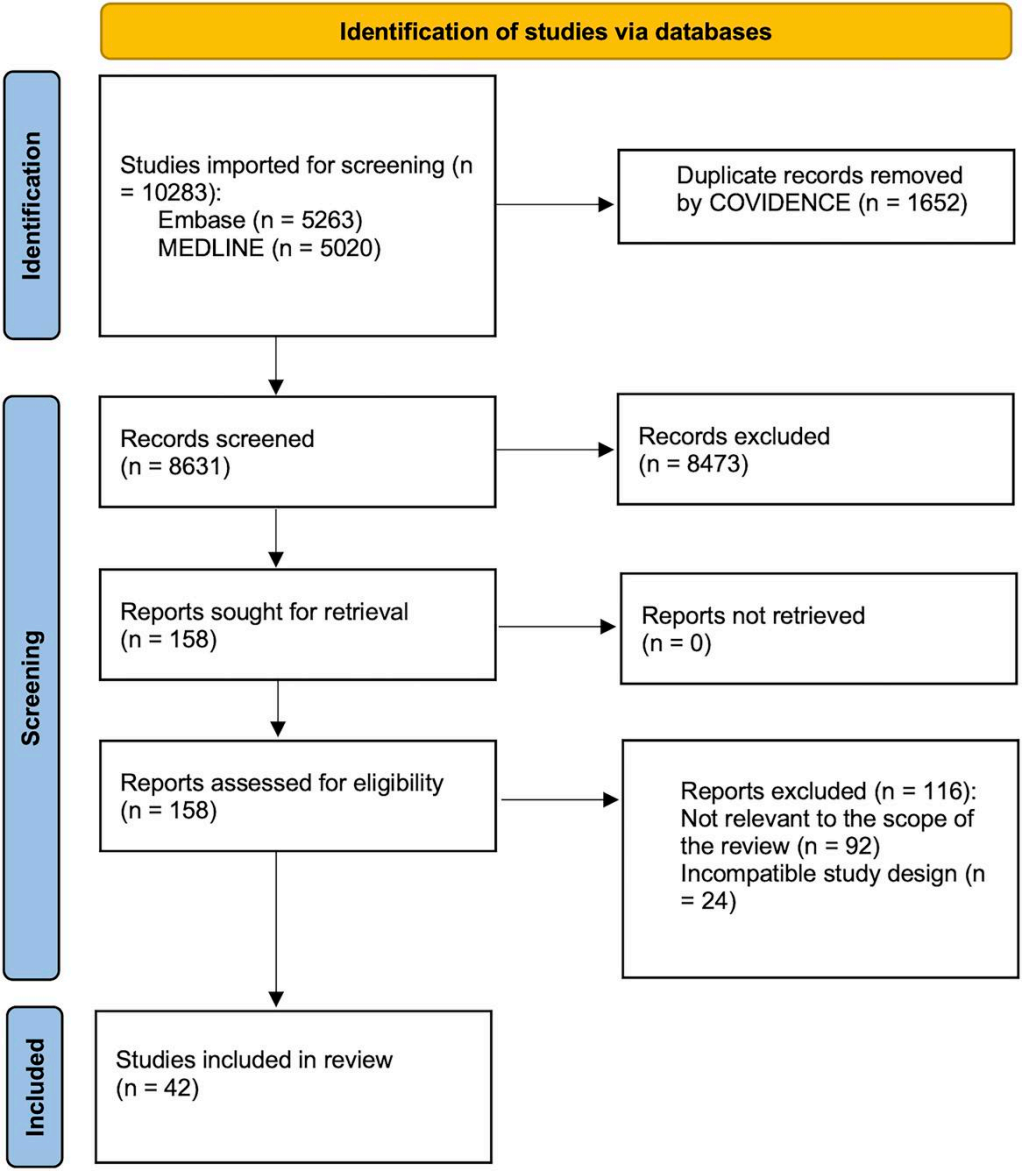
PRISMA-ScR flow diagram of study identification, screening, eligibility assessment, and inclusion for this scoping review. Literature searches of MEDLINE and EMBASE identified 10 283 records. After removal of duplicates and sequential title/abstract and full-text screening, 42 studies met inclusion criteria and were included in the final review.

All citations were imported into Covidence for deduplication and screening. A total of 10 283 records were identified, including 5020 records from MEDLINE and 5263 records from EMBASE. After removal of 1652 duplicate records, 8631 unique records remained for title and abstract screening. Two reviewers independently screened titles and abstracts, followed by full text review of potentially relevant studies, with disagreements resolved by consensus. The study selection process is summarized in *Figure 1*. Further details on article screening and selection are found in *Supplement S1*.

## Intravascular ultrasound

### IVUS technology, principles, and artifacts

**Figure 2:**
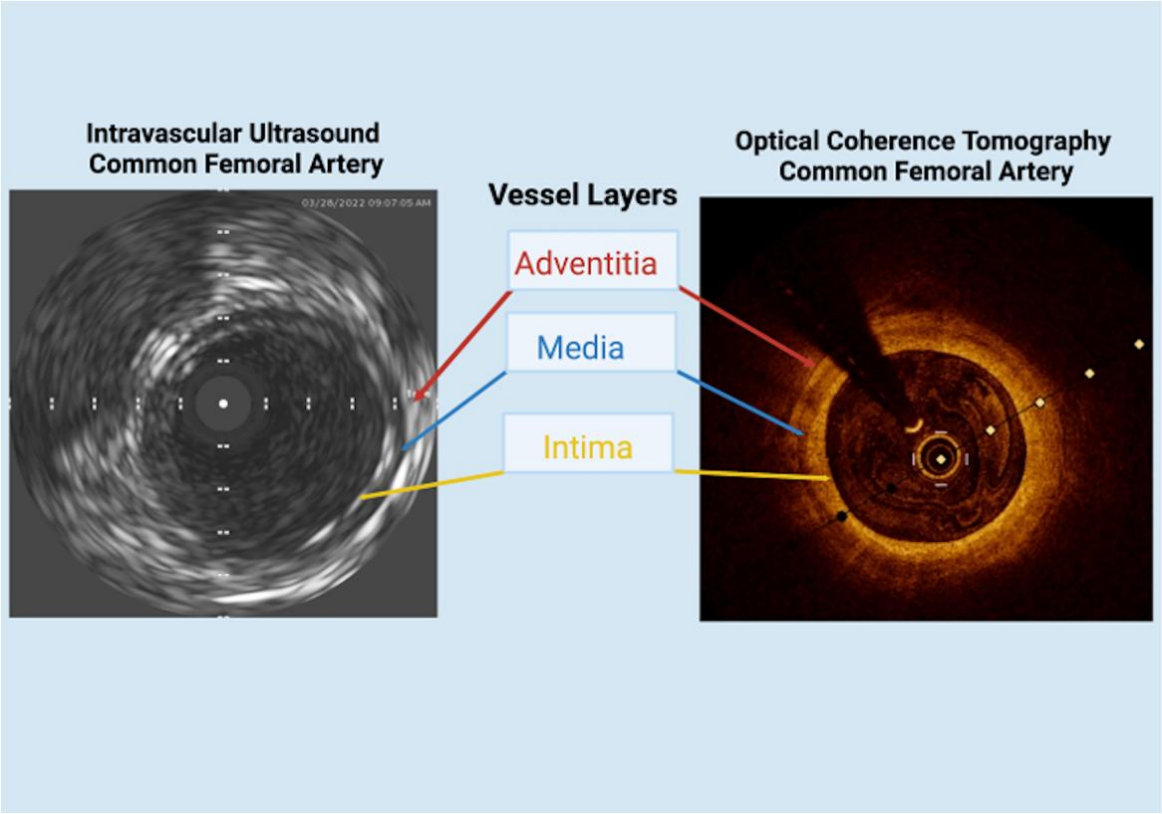
Both IVUS and OCT can be used to visualize vessel layers (intima, media, and adventitia), as well as any pathology present (i.e.: intimal thickening, fibrous or lipid plaques, calcification).

**Figure 3:**
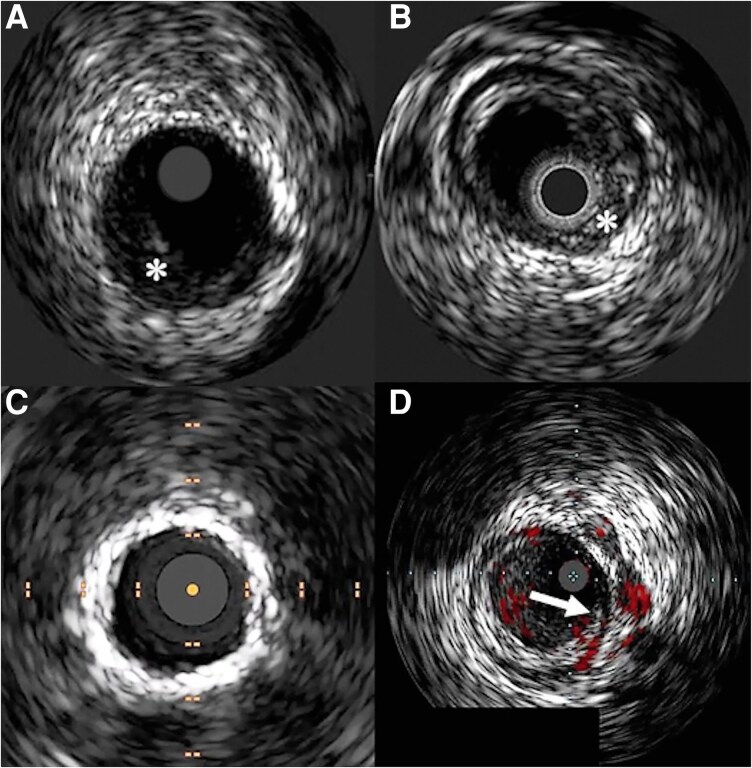
(*A*) Echolucent fibro-fatty plaque (asterisk), likely compliant lesion. (*B*) Echogenic fibrous plaque (asterisk). (*C*) Highly echogenic plaque with 360° of superficial calcium. (*D*) ChromaFlo being used on an IVUS image (arrow). Red indicates blood flow. Reproduced with permission from Medina *et al.* Intravascular Imaging for Peripheral Artery Disease and Endovascular Intervention of the Lower Extremities, Current Cardiovascular Imaging Reports, 2025.

IVUS utilizes piezo-electric crystals to generate ultrasound pulses under electric current, and transmitted pulses produce images in greyscale when transmitted back to the transducer, creating a cross-sectional image of the vessel. This cross-sectional image displays the intima, media and adventitial layers of the vessel (*Figure 2*), and readily identifies plaque burden, plaque morphology, and lumen diameter with higher fidelity than angiography.^20,21^ Bright signals are produced by more echogenic structures such as calcifications, whereas dark signals are generated by echolucent structures such as lipid collections.^21^  *Figure 3* shows examples of fibro-fatty (echolucent), fibrous (echogenic), and calcific plaques (highly echogenic). Higher frequency increases resolution but decreases depth of penetration.^21,22^

To obtain images, the IVUS catheter moves along the length of the segment of interest by manual or automatic pullback. The IVUS automatic pullback speed is generally between 0.5 mm/s and 1 mm/s, though some operators may perform manual pullback, especially in long lesions.^22,23^ If an automatic pullback is preferred, the ACIST (Minnesota, USA) Kodama IVUS catheter offers a faster automatic pullback speed of up to 10 mm/s.^24^ Additionally, ‘live’ imaging can clarify areas of uncertainty.^23^ Longer working lengths are required to reach distal peripheral imaging sites such as the tibial arteries. For these cases, longer imaging catheters, such as the Infraredex (Massachusetts, USA) Clarispro IVUS catheter, with a working length of 160 cm, may be utilized effectively.^25^ Peripheral IVUS catheters are available in both over-the-wire and rapid exchange systems. *Table 1* summarizes the commercially available peripheral IVUS catheters, including their minimum guide catheter size (Fr), working length (cm), transducer frequency, maximum imaging diameter (mm), format (over-the-wire or rapid exchange), and maximum guidewire size (inch).

**Table 1:** Commercially available peripheral IVUS catheters

Company	Device	Minimum guide catheter size (Fr)	Scanning mechanism (Solid state vs. rotational)	Format	Entry profile	Working length (cm)	Transducer frequency (MHz)	Maximum imaging diameter (mm)	Maximum guide wire size (inch)	Other
*Phillips*	Visions PV 0.14P RX	5	Solid state	Rapid Exchange		150	20	20	0.014	+ChromaFlo +Grayscale
	Visions PV 0.018 RX	6	Solid state	Rapid Exchange		135	20	24	0.018	+ChromaFlo +Grayscale
	Visions PV 0.35 OTW	8.5	Solid state	Over-the-wire		90	10	60	0.035	+Grayscale -Chromaflo
	Reconnaissance PV 0.18 OTW	5	Solid state	Over-the-wire		150	20	20	0.018	+Grayscale +ChromaFlo
	Pioneer Plus Re-entry	6	Solid state	Rapid Exchange (with OTW needle lumen)		120	20	20	0.014	24G Needle Depth: 3, 5, and 7 mm Needle guide wire: 0.014”
*Boston Scientific*	OptiCross 18	6	Rotational		1.6F	135	30	12	0.018	Typical use: SFA, Popliteal, Tibial, Renal
	OptiCross 35	8	Rotational		6F	105	15	70	0.035	Typical use: Iliacs, Femoral, and IVC
*ACIST*	Kodama	6		Rapid Exchange	1.7F	141	60	20	0.014	−+VariFlex -Max pullback speed 10 mm/sec
*Infraredex*	Clarispro HD-IVUS	6			2.4F	160	35–65	16	0.014	
	Dualpro	5			2.4	160	35–65	16	0.014	+NIRS

Commercially available IVUS catheters.

IVC, inferior vena cava; SFA, superficial femoral artery.

A key advantage of IVUS compared with digital subtraction angiography (DSA), the traditional gold standard for guiding PEI, is the ability to more accurately determine arterial dimensions^23,26–28^. IVUS also provides detailed information on lesion morphology and true lesion length, reducing the risk of geographic miss during treatment. Unlike DSA, which cannot directly visualize plaque, IVUS enables characterization of plaque composition and distribution. This has important clinical implications: a negatively remodelled artery may appear stenotic on DSA and be presumed to harbour plaque, while IVUS may reveal the absence of significant plaque burden.^23^ Conversely, IVUS may identify eccentric plaque, where one arterial wall segment is free of disease.^23^ In such cases, the selection of therapies such as atherectomy must be carefully considered to avoid vessel injury. Following stent implantation, IVUS can detect post-treatment arterial dissections with greater accuracy than DSA.^27^ Postangioplasty dissections have been associated with vessel patency outcomes.^29^ However, there is currently no consensus on whether dissections identified by IVUS should be treated.^30^

Technological differences exist between commercially available IVUS systems. Some Philips (Amsterdam, Netherlands) catheters incorporate ChromaFlo® imaging, which uses Doppler-based IVUS to visualize residual blood flow and can aid in identifying lumen size, dissections, thrombus, bifurcations, or incomplete stent apposition.^31^ This feature is not available on Boston Scientific (Marlborough, Massachusetts) IVUS catheters, which instead emphasize optimized resolution and deliverability.^32^  *Figure 3* shows example IVUS images in PEI. Both platforms are subject to common ultrasound artefacts, including ring-down (a bright circumferential echo near the catheter tip), which must be recognized to avoid misinterpretation.^21^

#### Limitations of IVUS

Additional ultrasound artefacts can occur, including reverberation and acoustic shadowing. In mechanical rotational probes, non-uniform rotational distortion (NURD) may also arise, typically caused by asymmetric friction or catheter torsion. Air bubbles can create artefacts by reflecting ultrasound waves before they reach tissue structures. Catheter flushing is therefore essential, and should be done outside the body to prevent air embolism.^21^

### IVUS clinical evidence and outcomes

Despite the potential advantages of IVUS over DSA in PEI, high-quality prospective data is limited.^33^ Available evidence suggests that IVUS-guidance during PEI can reduce restenosis rates and improve primary patency rates, especially when used with drug-coated balloons (DCB)^34–36^. Some data suggest that IVUS-guided PEI, including stenting of chronic total occlusions, compared to no IVUS, is associated with decreased major adverse limb events (MALE), notably lower amputation rates^37–44^. In contrast, certain studies have not demonstrated a beneficial effect of IVUS utilization in PEIs, particularly when combined with stent implantation.^43,45^ Although not yet included in major guidelines, expert consensus suggests added value of IVUS while evaluating peripheral vessel occlusions and optimizing PEI in both arterial and venous beds.^46^ This is evidenced by the growing adoption of IVUS in PEIs, especially in ambulatory surgery centres (ABS) and office-based laboratories (OBL).^4,37,38^

Despite growing evidence supporting the clinical and economic value of IVUS-guided PEIs, its widespread adoption remains constrained by reimbursement policies and upfront cost considerations that vary substantially across healthcare systems. A recent Japanese analysis by Yoshimitsu Soga *et al.* demonstrated that IVUS-guided peripheral revascularization was associated with a mean cost saving of approximately $18 000 per patient over follow-up, largely driven by reductions in repeat revascularization and major adverse cardiac and limb events.^47^ However, realization of these long-term cost benefits depends on reimbursement frameworks that adequately support the initial procedural and equipment costs of IVUS.

Coverage for IVUS outside the USA remains inconsistent, which has limited its broader international adoption and standardization of practice, as highlighted by the Society for Cardiovascular Angiography and Interventions expert consensus.^11^ Even within the USA, current diagnosis-related group-based reimbursement models may disincentivize the routine use of IVUS by failing to account for downstream cost savings, thereby placing financial pressure on institutions despite potential improvements in patient outcomes.^11^ Consequently, reimbursement and economic considerations represent a key barrier to the global dissemination of IVUS, underscoring the need for payment models that better align short-term procedural costs with long-term clinical and economic value.

To date, there have been two RCTs investigating the use of IVUS in PEI. An RCT from South Korea compared IVUS-guided (*n* = 119) vs. angiography-guided (*n* = 118) DCB of femoropopliteal artery (FPA) disease. At 12 months, primary patency was higher with IVUS (83.8% vs. 70.1%, *P* = 0.01).^35^ IVUS use was associated with larger balloon diameters, resulting in greater postprocedural minimum lumen diameter (3.9 mm vs. 3.71 mm, *P* = 0.03).^35^ Secondary safety endpoints (all-cause death, cardiovascular death, major bleeding) did not differ. A prespecified subgroup analysis of this study focusing on complex FPA lesions (defined as Trans-Atlantic Inter-Society Consensus (TASC) II type C or D lesions) showed that IVUS improved primary patency, freedom from clinically driven target vessel revascularization (CD-TLR), and sustained clinical and haemodynamic improvement.^36^ An RCT from Australia and New Zealand compared IVUS (*n* = 76) vs. angiography (*n* = 74) in FPA PEI.^34^ In the IVUS group, 57.9% received DCB, 31.5% stents (bare-metal stent [BMS], covered stent, or drug-eluting stent [DES]), and 10.5% plain-old balloon angioplasty (POBA). In the angiography group, 54% received DCB, 31.1% stents, and 14.9% POBA. IVUS significantly reduced restenosis (55.4% vs. 72.4%, *P* = 0.008). Sub-analysis showed the benefit was driven by DCB cases, while IVUS-guided stenting did not improve outcomes.^48^ Overall, RCT data support IVUS-guided PEI with DCB, while evidence for IVUS-guided POBA or stenting remains inconclusive. The benefit appears particularly greater in complex lesions (TASC II type C/D). *Table 2* summarizes these two RCTs.

**Table 2:** Randomized controlled trials using IVUS vs. angiography in peripheral interventions

	Number of patients	Lesion details	Study population and treatment	Follow up (days)	Restenosis rate- IVUS	Restenosis rate- angiography	*P* value	Comments
Allan *et al.* (2022)	Total: 150 (IVUS: 76) (angiography:74)	De novo stenosis, CTOs, and restenotic FPA lesions (SFA and popliteal artery); PACSS (angiography vs. IVUS): 0 (23 vs. 30), 1 (15 vs. 15), 2 (1 vs. 5), 3 (4 vs. 4), 4 (31 vs. 22).	Patients undergoing FPA interventions IVUS Group: DCB angioplasty: 57.9% Stenting: 31.5% (either BMS, covered stenting, or DES) POBA: 10.5% angiography Group: DCB angioplasty: 54% Stenting: 31.1% (either BMS, covered stenting, or DES) POBA: 14.9%	365	55.4%	72.4%	0.008	Binary restenosis significantly lower in IVUS group for cases treated with DCB
Ko *et al.* (2024)	Total: 237 (IVUS: 119) (angiography:118)	Symptomatic FPA lesions treated with DCB angioplasty; lesion complexity and morphology by group: TASC A–C vs. D (angiography: 63 vs. 55; IVUS: 60 vs. 59), CTO (angiography: 68 [58.1%] vs. IVUS: 78 [66.7%]), calcification by PACSS grouped categories: 0–2 (angiography: 72 vs. IVUS: 65) and 3–4 (angiography: 46 vs. IVUS: 54).	Patients undergoing FPA interventions with DCB	363	16.2%	29.9%	0.01	Freedom from TLR and sustained clinical and hemodynamic improvement significantly higher in IVUS group

RCTs to date examining IVUS use in peripheral interventions.

BMS, Bare-metal stent; DCB, Drug-coated balloon; DES, Drug-eluting stent; FPA, Femoropopliteal artery; IVUS, Intravascular ultrasound; PACSS, peripheral arterial calcium scoring system; POBA, Plain old balloon angioplasty; TASC, TransAtlantic InterSocietal Consensus; TLR, Target lesion revascularization.

Three meta-analyses have investigated the role of IVUS in PEI. In 2020, Sheikh *et al.* reported no significant differences in rates of primary patency, reintervention, and amputations with IVUS use compared to no IVUS. However, the rate of periprocedural adverse events and vascular complications were significantly lower in the IVUS group.^49^ In 2024, Tsukagoshi *et al.* refuted some of the previously reported results by Sheikh *et al.* After including a more recent RCT and one of the largest cohort studies to date in their meta-analysis, Tsukagoshi *et al.* found a significantly higher primary patency and lower risk of major amputation in those undergoing IVUS guided PEI for FPA disease.^34,37,42^ Most recently, Jang *et al.* performed a meta-analysis which included 800 452 patients from 19 studies, including the two previously discussed RCTs by Ko *et al.* and Allan *et al.*^17^ Compared with DSA-only guided PEI, IVUS significantly reduced the risk of binary restenosis (RR 0.63, 95% CI 0.43–0.91, *P* = 0.02).^17^ IVUS-guided PEI was also associated with improved outcomes across all secondary endpoints, including reintervention, major amputation, death or amputation, and major adverse limb events (MALE), compared with DSA guidance alone.^17^

Considering that IVUS use is not yet in the guidelines and recognizing the need for appropriate use criteria, Secemsky *et al.* published an expert consensus opinion on the appropriate use of IVUS during arterial and venous lower extremity interventions.^46^ The writing committee graded IVUS as ‘appropriate’ for all levels of PAD revascularization.^50^  *Tables 3–6* summarize recent studies from 2004–2024, including review articles, retrospective reviews, meta-analyses, and prospective observational and RCTs that have evaluated the use of IVUS in PEIs, as well as treatment with POBA, DCB, and stenting (BMS, covered stent, and DES).

**Table 3:** IVUS use in peripheral arterial interventions (2006–2010)

Study	Number of patients	Lesion details	Study population	Follow-up	IVUS type	IVUS outcomes	No-IVUS outcomes	*P* value	Other findings
Jacobs *et al.* (2006), retrospective	22 patients (24 CTO); IVUS-guided re-entry: 20 patients, fluoroscopy-guided re-entry: 2 patients.	87 CTO lesions of the common iliac, external iliac, combined common–external iliac, bilateral iliac, and femoral occlusions, including long femoral lesions ≥10 cm. Mean occlusion length: iliac 8.1 cm, femoral 13.5 cm. Lesion complexity predominantly TASC B–D.	Patients who underwent treatment of iliac or SFA CTO with **POBA or SI**	5.8 months	Pioneer dual lumen phased array IVUS (re-entry device)	IVUS guided lumen re-entry was successfully used in 21 of the lesions, and the other 3 lesions cross with angiographic guidance using an Outback catheter	In 24/87 lesions (18 iliac, 3 SFA), the true lumen could not be reentered with conventional techniques	n/a	All patients had an increase in their ABIs by at least 0.1 Mean ABI at 6 months increased from 0.58 pre procedure to 0.97 post procedure All occlusions treated with IVUS guided true lumen re-entry demonstrated clinical success at follow up
Kawasaki *et al.* (2008), retrospective	47 patients	52 CTO lesions of the FPA and iliac arteries (phase 1: *n* = 21; phase 2: *n* = 31).; lesion complexity by TASC classification: iliac lesions phase 1 A/B/C/D = 0/4/2/1 and phase 2 = 0/2/1/5; FPA lesions phase 1 = 2/3/3/6 and phase 2 = 2/6/10/5. Mean occlusion length (mm): iliac 47 ± 41 vs. 68 ± 39; FPA 141 ± 101 vs. 82 ± 72. Angiographic calcification present (iliac: 5 vs. 6; FPA: 5 vs. 12).	Patients with lower extremity CTO of the iliac (15) and/or FPA (37) who were treated with **SI**	n/a	Eagle Eye Gold phased array IVUS	97% technical success rate with ABI 1.0 +/− 0.14	81% technical success rate with post procedural ABI 0.89 +/− 0.22	<0.01	Lowest contrast use in IVUS group Stent size larger in no IVUS group
Krishnamurthy *et al.* (2010), retrospective	11 patients	Unilateral iliac artery chronic total occlusions; lesion location: common iliac artery (*n* = 7), external iliac artery (*n* = 1), combined common and external iliac artery (*n* = 3). Lesion length 3–15 cm (mean 5.2 ± 1.1 cm). Lesion complexity by TASC classification: TASC B (*n* = 8) and TASC D (*n* = 3).	Patients with CTO of the iliac arteries who failed conventional subintimal recanalization who then underwent IVUS guided **SI**	10.5 months	Pioneer dual lumen phased array IVUS (re-entry device)	100% technical success rate and improvement of symptoms at follow up	Patients who failed conventional subintimal recanalization were included, but not compared	n/a	All patients had palpable femoral pulses and normalization of ABI at follow up Re entry was achieved in < 10 min in all patients No procedure related complications

Studies investigating IVUS use in peripheral arterial interventions (2004–2010).

ABI, ankle-brachial index; CTO, chronic total occlusion; FPA, femoropopliteal artery; IVUS, intravascular ultrasound; PACSS, peripheral arterial calcium scoring system; POBA, plain old balloon angioplasty; SFA, superficial femoral artery; SI, stent implantation; TASC, TransAtlantic InterSocietal Consensus.

**Table 4:** IVUS use in peripheral arterial interventions (2011–2015)

Study	Number of patients	Lesion details	Study population	Follow-up	IVUS Type	IVUS outcomes	No-IVUS outcomes	*P* value	Other findings
Kawasaki *et al.* (2011), retrospective	Thirty six patients with chronic renal insufficiency.	Iliofemoral artery lesions (*n* = 51); iliac artery lesions (*n* = 32): TASC A (*n* = 16), B (*n* = 4), C (*n* = 8), D (*n* = 4), including chronic total occlusions (*n* = 8); femoral artery lesions (*n* = 19): TASC A (*n* = 4), B (*n* = 3), C (*n* = 7), D (*n* = 5), including chronic total occlusions (*n* = 11).	Patients femoral (19) and/or iliac (32) artery stenosis and with CKD undergoing **balloon dilatation with or without SI** IVUS guided without contrast	Ten months	Eagle Eye Gold phased array IVUS	Mean ABI increased from 0.59 to 0.92 and was maintained for 3 months	n/a	n/a	No post procedural deaths, strokes of amputations Demonstrated feasibility of zero contrast endovascular therapy in patients at high risk for renal failure
Ciopa *et al.* (2012), retrospective	30 patients	Severely calcified femoropopliteal artery lesions (*n* = 30) treated with directional atherectomy and drug-coated balloon; lesion location: proximal SFA (*n* = 5), mid SFA (*n* = 15), distal SFA (*n* = 7), popliteal artery (*n* = 3). Total occlusions (*n* = 4). Mean lesion length 115 ± 35 mm. All lesions with heavy calcification (calcium score ≥3). TASC II type A–C lesions only.	Patients with FPA lesions with life limiting claudication or critical limb ischaemia who underwent IVUS guided atherectomy and **DCB**	12 months	n/a	100% technical success with primary patency rate of 90% at 1 year	n/a	n/a	No major amputations ABI at one year was improved from 0.5 to 0.8 at 12 month follow up
Araki *et al.* (2013), retrospective	82 patients	Iliac artery chronic total occlusions (*n* = 86); lesion location: common iliac artery (*n* = 51), external iliac artery (*n* = 19), combined common and external iliac artery (*n* = 16). Lesion complexity by TASC II: type B (*n* = 34), type C (*n* = 22), type D (*n* = 30). Heavy calcification on IVUS in *n* = 27 lesions. Mean occlusion length 6.5 ± 2.69 cm.	Patients with CTO of the iliac artery who underwent *self-expandable stent placement*	27.6 months		Primary patency rate of 95.9% at 2 years	n/a	n/a	No episodes of peripheral embolization or iliac artery rupture Maximum luminal diameter was increased at follow up angiography compared to immediately post procedure
Iida *et al.* (2014), retrospective, PSM	965 patients with 1198 limbs. PSM into 234 IVUS, 234 No-IVUS	FPA lesions limited to TASC II A–C; TASC distribution in overall cohort: A (*n* = 448), B (*n* = 440), C (*n* = 310). Mean lesion length 88 ± 49 mm in non-IVUS group vs. 104 ± 49 mm in IVUS group. Angiographic calcification present in non-IVUS group (*n* = 513) and IVUS group (*n* = 114). Chronic total occlusions included; TASC D lesions excluded.	Patients undergoing FPA nitinol **SI**	1.9 years	n/a	65%±6% primary patency	35%±6% primary patency	<0.001	IVUS resulted in significantly greater freedom from any reintervention and event-free survival
Baker *et al.* (2015), retrospective	20 (IVUS) 19 (Matched No-IVUS Cohort)	Lower extremity CTO lesions (attempted *n* = 20; successfully treated *n* = 18) involving the common iliac artery (*n* = 10), external iliac artery (*n* = 3), and superficial femoral artery (*n* = 5); all lesions TASC II B or higher, including TASC C–D disease, treated with subintimal angioplasty and IVUS-guided true lumen reentry.	Patients undergoing CTO intervention of CIA, EIA, and SFA with **SI**: DES (1), BMS (4), Covered Stent (6), Uncovered Stent (7), Unsuccessful (2)	4 months (IVUS) 10 months (Matched Cohort)	IVUS-RED	62% primary patency	71% primary patency	0.82	No significant difference in SVS runoff scores between two groups

Studies investigating IVUS use in peripheral arterial interventions (2011–2015).

ABI, ankle-brachial index; BMS, bare-metal stent; CIA, common iliac artery; CTO, chronic total occlusion; DCB, drug-coated balloon; DES, drug-eluting stent; EIA, external iliac artery; EVT, endovascular therapy; FPA, femoropopliteal artery; IVUS, intravascular ultrasound; PSM, propensity score matching; SFA, superficial femoral artery; SI, stent implantation; SVS, Society of Vascular Surgery; TASC II, Trans-Atlantic Inter-Society Consensus II.

**Table 5:** IVUS use in peripheral arterial interventions (2015–2020)

Study	Number of patients	Lesion details	Study population	Follow-up	IVUS type	IVUS outcomes	No-IVUS outcomes	*P* value	Other findings
Kamakura (2015), observational	455 patients	507 De novo iliac artery lesions treated with IVUS-guided primary: common iliac artery (*n* = 201), external iliac artery (*n* = 240), combined common–external iliac artery (*n* = 54), aortoiliac artery (*n* = 12). Lesion complexity by TASC II: A/B (*n* = 364) and C/D (*n* = 143). CTOs (*n* = 119). Calcified lesions identified by IVUS (*n* = 97). Mean lesion length 5.8 ± 4.9 cm.	Patients with severe iliac artery stenosis who underwent primary **SI**	15 years	IVUS catheter (Visions PV 0.018, 20 MHz, Volcano Corp., or Ultracross catheter, 30 MHz, Boston Scientific)	5,10, 15-year primary patency rates were 89%, 83,%, and 92%, respectively	n/a	n/a	post-procedural MLA, in-stent thrombosis, discontinuation of antiplatelet therapy, and calcified lesions were independent predictors of primary patency.
Panaich *et al.* (2016), retrospective	92 714 patients total; 1299 IVUS, 91 415 No-IVUS.	n/a	Patients undergoing lower limb endovascular interventions	n/a	n/a	12% any complication or death	14.9% any complication or death	<0.001	IVUS use lead to lower amputation rates and non-significant increased hospitalization cost
Krishnan *et al.* (2018), retrospective	114 patients; 46 IVUS, 68 No-IVUS	114 FPA in-stent restenosis lesions treated with directional atherectomy; ISR classification: class I (*n* = 24), class II (*n* = 61), class III (*n* = 29). CTOs present (IVUS *n* = 14; angiography *n* = 15). Mean lesion length 16.61 ± 8.3 cm (IVUS) vs. 1.67.5 ± 6.63 cm (angiography). Calcification present (IVUS *n* = 5; angiography *n* = 3). Iliac disease excluded.	Patients undergoing directional atherectomy plus POBA for FPA in-stent restenosis	12 months	n/a	17.9% clinically driven TLR	51% clinically driven TLR	0.03	No cases of thrombosis, perforation, amputation, access site complications or death in both groups
Sheikh *et al.* (2020), systematic review and meta-analysis	93 551 patients total; 1733 IVUS; 91 818 No-IVUS.	n/a	Patients undergoing peripheral vascular interventions in the iliac, femoral or popliteal arteries	24 months	n/a	Risk Ratios (IVUS vs. no-IVUS) Primary Patency: 1.3 (0.99–1.71, *P* = 0.062) Reintervention: 0.41 (0.15–1.13, *P* = 0.085) Amputation: 0.83 (0.32–2.15, *P* = 0.705)			Rates of periprocedural adverse events and vascular complications were significantly lower in the IVUS group
Buckley *et al.* (2022), retrospective	52 patients with 71 limbs; 36 patients/49 limbs IVUS, 16 patients/22 limbs No-IVUS	71 Symptomatic aortoiliac occlusive disease treated with balloon angioplasty and primary stenting; lesion location: common iliac artery and/or external iliac artery. Lesion type included hemodynamically significant iliac stenoses (*n* = 64 limbs) and iliac CTOs (*n* = 7 limbs).	Patients with symptomatic aortoiliac disease underwent POBA + **SI**	62 months	20-MHz or 30-MHz ultrasound transducer	100% and 100% primary patency at 3 and 6 years, respectively	82% and 69% primary patency at 3 and 6 years, respectively	<0.001	No reinterventions in IVUS group compared to 23% reintervention in non-IVUS group
Tsujimura *et al.* (2022), PSM	1091 patients total; 843 IVUS; 248 No-IVUS	Symptomatic FPA lesions (SFA and proximal popliteal artery) treated with fluoropolymer-based DES; lesion length 1.93 ± 0.99 cm (IVUS) vs. 1.62 ± 0.95 cm (non-IVUS). CTOs present (IVUS *n* = 486; non-IVUS *n* = 103). Arterial calcification morphology: none (*n* = 357), unilateral wall calcification (*n* = 275), bilateral wall calcification (*n* = 459). Popliteal involvement (IVUS *n* = 193; non-IVUS *n* = 65).	Patients undergoing FPA interventions with fluoropolymer-based **DES**	12 months	n/a	11.5% rate of stenosis	15.5% rate of stenosis	0.22	Higher frequency of aneurysmal degeneration in IVUS group Less restenosis occurrence in patients with CTO

Studies investigating IVUS use in peripheral arterial interventions (2015–2020).

ABI, ankle–brachial index; CLI, Critical limb ischaemia; CTO, chronic total occlusion; DA, directional atherectomy; DCB, drug-coated balloon; DES, drug-eluting stent; EVT, endovascular therapy; ISR, in-stent restenosis; IVUS, intravascular ultrasound; PAD, peripheral artery disease; PTA, percutaneous transluminal angioplasty; RED, reentry device (IVUS-guided); SFA, superficial femoral artery; SI, stent implantation; TASC II, TransAtlantic Inter-Society Consensus II; TLR, target lesion revascularization.

**Table 6:** IVUS use in peripheral arterial interventions (2022–2025)

Study	Number of patients	Lesion details	Study population	Follow-up	IVUS type	IVUS outcomes	No-IVUS outcomes	*P* value	Other findings
Allan *et al.* (2022), RCT	150 patients; 76 IVUS, 74 No-IVUS	Symptomatic FPA lesions (SFA and popliteal artery). Angiography group (*n* = 74): lesion location SFA (*n* = 32), SFA + popliteal (*n* = 29), popliteal alone (*n* = 13); lesion type stenosis (*n* = 40), CTO (*n* = 24), restenosis (*n* = 10); PACSS calcification score 0 (*n* = 23), 1 (*n* = 15), 2 (*n* = 1), 3–4 (*n* = 35). IVUS group (*n* = 76): lesion location SFA (*n* = 35), SFA + popliteal (*n* = 26), popliteal alone (*n* = 15); lesion type stenosis (*n* = 41), CTO (*n* = 26), restenosis (*n* = 9); PACSS calcification score 0 (*n* = 30), 1 (*n* = 15), 2 (*n* = 5), 3–4 (*n* = 26).	Patients undergoing FPA interventions	12 months	Atlantis rotating mechanical IVUS (Boston Scientific) or Eagle Eye or PV018 phased array IVUS (Philips Volcano)	55.4% rate of restenosis	72.4% rate of restenosis	<0.01	Change in treatment due to IVUS findings occurred in 79% of cases Average DCB size was larger when used in the IVUS group, and restenosis rates were also lower compared to angiography alone
Divakaran *et al.* (2022), retrospective, PSM	543 488 Patient;63 732 IVUS, 479 759 No-IVUS	n/a	Patients >65 years old undergoing lower extremity PEI	514 days	n/a	14.1% rate of male	16.6% rate of male	<0.0001	Rates of ALI and major amputations lower with IVUS
Kurata *et al.* (2022), retrospective	165 patients; 231 lesions	FPA lesions treated with DCB; lesion complexity by TASC II: A (*n* = 82), B (*n* = 44), C (*n* = 60), D (*n* = 45). CTOs present (*n* = 42). Mean lesion length 13.3 ± 9.3 cm.	Patients with symptomatic PAD undergoing femoropopliteal EVT using IN.PACT Admiral DCB with routine IVUS evaluation	Mean 17 ± 9 months	AltaView IVUS (Terumo); automated pullback 9 mm/s	19.7 rate of restenosis	34.5 rate of restenosis	<0.05	DCB sizing based on **IVUS-EEM diameter** associated with significantly lower 2-year restenosis Angio-lumen–based and IVUS-lumen–based sizing did not reduce restenosis; no increase in severe dissections with larger IVUS-EEM–based DCB sizing
Smith *et al.* (2023), retrospective	65 038 patients; 3424 IVUS, 61 614 No-IVUS	Lesion complexity by TASC classification (overall cohort): TASC A (*n* = 12 445), B (*n* = 15 150), C (*n* = 11 526), D (*n* = 9114), unknown (*n* = 16 803).	Patients undergoing first-time FPA endovascular revascularization for chronic occlusive PAD.	12 months	n/a	83.7% rate of amputation-free survival	80.2% rate of amputation-free survival	<0.01	IVUS use increased 13-fold during study period (0.6% → 8.2%); adoption concentrated in ambulatory centres. Benefits may be confounded by healthier baseline population.
Setogawa *et al.* (2023), retrospective, PSM	85 649 patients; 50 925 IVUS, 34 724 No-IVUS	n/a	Patients undergoing lower extremity PEI (angioplasty and/or stenting)	12 months	n/a	5% rate of amputation	10.7% rate of amputation	<0.01	IVUS group had lower risk of bypass surgery, stent grafting, and decreased hospital costs
Krishnan *et al.* (2023), retrospective	200 patients total; 91 IVUS, 109 No-IVUS	mean lesion length 9.06 ± 4.17 cm (angiography) vs. 11.75 ± 4.93 cm (IVUS). CTOs present (angiography *n* = 46; IVUS *n* = 32). Calcification present (angiography *n* = 14; IVUS *n* = 35). Dissection more frequent in IVUS group (angiography *n* = 20; IVUS *n* = 48).	PAD patients with ≥1 SUPERA stent (236 stents total); IVUS was used for present sizing and deployment guidance	3 years	Philips Volcano Eagle Eye (0.014’) or PV018 (0.018’) phased-array catheters	68% rate of nominal deployment	38% rate of nominal deployment	<0.001	Reintervention lower with IVUS No difference in amputation or mortality.
Soga *et al.* (2023), retrospective	9845 patients; 3.956 IVUS, 5889 No-IVUS	n/a	Patients undergoing lower extremity PEI (angioplasty and/or stenting)	Variable	n/a	55% rate of MACLE	65% rate of MACLE	<0.0001	IVUS significantly reduced rate of stroke, and re-interventions. Cost reduction of $18, 173 per patient with IVUS
Zou *et al.* (2023), retrospective	137 patients; 43 IVUS, 94 No-IVUS	Symptomatic FPA lesions. Angiography group: stenosis (*n* = 27), CTO (*n* = 67); TASC II B (*n* = 10), C (*n* = 45), D (*n* = 39); calcification none (*n* = 23), mild (*n* = 53), severe (*n* = 18); median lesion length 16.1 cm. IVUS group: stenosis (*n* = 16), CTO (*n* = 27); TASC II B (*n* = 9), C (*n* = 18), D (*n* = 16); calcification none (*n* = 8), mild (*n* = 22), severe (*n* = 13); median lesion length 16.2 cm.	Patients with symptomatic PAD involving the FPA mostly with moderate–severe disease (Rutherford 3–5).	12 months	40-MHz IVUS (Boston Scientific OptiCross); automated pullback 1 mm/s	76.7% rate of primary patency	56.4% rate of primary patency	<0.05	IVUS revealed larger reference diameters, guided balloon sizing, and detected more dissections. Predictors of restenosis: longer lesions ↑ risk; higher balloon-to-vessel ratio protective.
Tsukagoshi *et al.* (2024), systematic review and meta-analysis	708 808 patients 709 189 lesions; 101 405 IVUS, 607 784 No-IVUS	Varied per study	Patients undergoing IVUS-guided peripheral interventions	Varied per study	Varied per study	Risk ratios (IVUS vs. no-IVUS) Restenosis/occlusion: 0.74 (0.0.54–1.00, *P* =nonsignificant) Reintervention or all-cause mortality: 0.0.85 (0.65–1.1, *P* = nonsignificant) Amputation: 0.74 (0.67–0.82, *P* = significant)			The use of IVUS was associated with significantly lower risk of amputation
Kumar *et al.* (2024), retrospective	434 901 patients; 9969 IVUS, 424 932 No-IVUS	n/a	Patients from the National Readmission database who received PEI for PAD between January 2016-December 2019	6 months	n/a	2.17% amputation rate	2.71 amputation rate	Non-significant	Subgroup of patients with rest pain, iliac intervention, or DES placement had significantly lower amputation rates with IVUS
Kodama *et al.* (2024), retrospective	2227 patients; 784 IVUS, 1443 No-IVUS	n/a	Consecutive patients from TOMACODE	10.4 months	n/a	1.6% in-hospital MALE	4.1% in-hospital MALE	0.01	IVUS group had higher procedural success rate
Ko *et al.* (2024), RCT	237 patients; IVUS: 119, No-IVUS:118)	Symptomatic FPA lesions (SFA and proximal popliteal artery) treated with DCB angioplasty; lesion length 20.5 ± 10.3 cm (IVUS) vs. 21.5 ± 10.3 cm (angiography). CTOs present (IVUS *n* = 78; angiography *n* = 68). Lesion complexity by TASC II: A–C (IVUS *n* = 60; angiography *n* = 63) and D (IVUS *n* = 59; angiography *n* = 55). Calcification severity by PACSS: 0–2 (IVUS *n* = 65; angiography *n* = 72) and 3–4 (IVUS *n* = 54; angiography *n* = 46).	Patients undergoing FPA artery interventions with DCB	363 days	Volcano s5 (Philips) or iLab2 (Boston Scientific)	16.2% restenosis	29.9% restenosis	0.01	Freedom from TLR and sustained clinical and hemodynamic improvement significantly higher in IVUS group
Jang *et al.* (2025), meta-analysis	800 452 patients; 125 254 IVUS, 675,198, No-IVUS	Varied per study	Patients undergoing IVUS-guided peripheral interventions	Varied per study	Varied per study	Risk ratios (IVUS vs. no-IVUS) A restenosis/occlusion: 0.63 (0.43–0.91, *P* = 0.02, significant) Reintervention: 0.59 (0.39–0.90, *P* = 0.01, significant) All-cause death or amputation: 0.72 (0.56–0.91, *P* = 0.007, significant) Amputation: 0.85 (0.74–0.98, *P* = 0.02, significant) MALE: 0.52 (0.28–0.94, *P* = 0.03, significant)			Largest evidence pool to date; demonstrates broad benefit of IVUS guidance across endpoints. Subgroup (femoropopliteal only): same consistent benefits

Studies investigating VUS use in peripheral arterial interventions (2022–2024).

ALI, acute limb ischaemia; DCB, drug-coated balloon; DES, drug-eluding stent; EVT, endovascular therapy; FPA, femoropopliteal artery IVUS, intravascular ultrasound; MALE, major adverse limb events; MACLE, major adverse cardiac and limb events; PAD, peripheral arterial disease; PACSS, peripheral arterial calcium scoring system; PEI, peripheral endovascular intervention; PSM, propensity score matching; RCT, randomized-controlled trial; TASC II, TransAtlantic Inter-Society Consensus II; TLR, target-lesion revascularization; TOMA-CODE Tokyo Tama peripheral vascular intervention research comrade.

### Optical coherence tomography (OCT)

#### OCT technology, principles, and artifacts

OCT utilizes near-infrared light to create high-resolution cross-sectional images of blood vessels. Like IVUS, the cross-sectional image displays the intima, media and adventitial layers of the vessel (*Figure 2*). OCT emits light that is absorbed, backscattered, or reflected by tissue structures, forming images based on the magnitude and time delay of the reflected light.^51^  *Table 7* summarizes the commercially available OCT catheters, including their minimum guide catheter size (Fr), working length (cm), maximum guide wire size (inch) and crossing profile (mm).

**Table 7:** Commercially available peripheral OCT guided atherectomy and imaging catheters

Company	Device	Minimum guide catheter size (Fr)	Working length (cm)	Maximum guide wire size (inches)	Maximum imaging diameter (mm)	Crossing profile
Avinger	Ocelot	6	110	0.014		2 mm
	Ocelot PIXL	5	135	0.014		1.7 mm
	Ocelot MVRX	6	110	0.014		2 mm
	Pantheris	7	110	0.014		0.100” or 0.110”*
	Pantheris Small Vessel	6	140	0.014		0.086”
	TigerEye	5	140	0.35		1.8 mm
Gentuity	HF-OCT Imaging System	6	165	0.014	14.5 mm	0.33 mm

Commercially available OCT catheters.

*Crossing profile varies by Pantheris device configuration.

**Figure 4:**
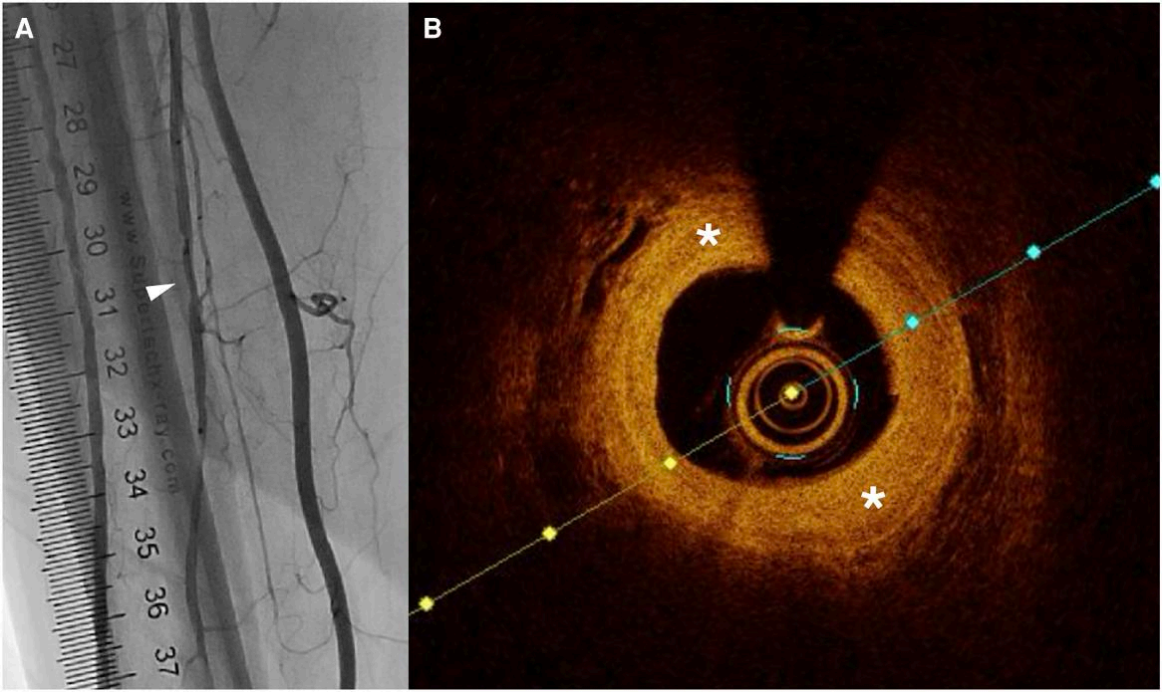
(*A*) Angiography of peroneal artery. The white arrowhead on the left panel of A denotes coregistration of the angiographic position to the right panel OCT cross-sectional image (*B*) OCT identified fibrotic plaque (white asterisk) with bright signal rich homogenous.

**Figure 5:**
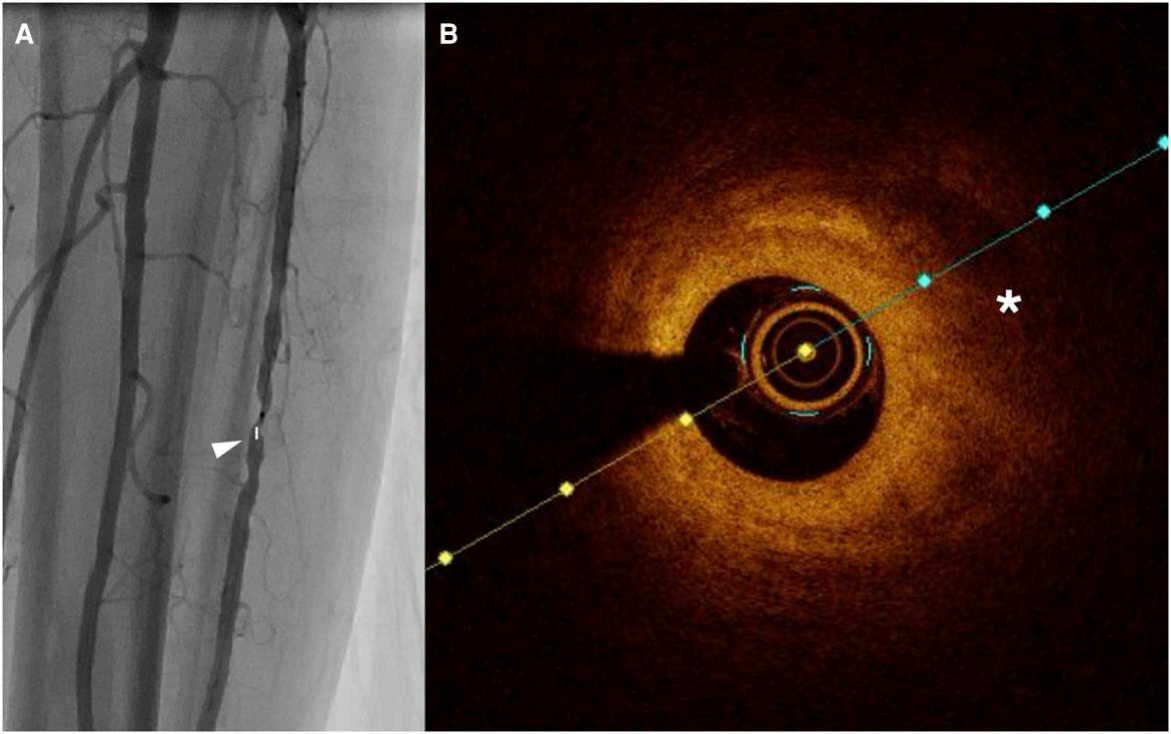
(*A*) Angiography of anterior tibial artery. The white arrowhead on the left panel of A denotes coregistration of the angiographic position to the right panel OCT cross-sectional image (*B*) OCT identified soft plaque with lipid (white asterisk). Lipid plaque identified by poor signal, low backscattering, and diffuse.

**Figure 6:**
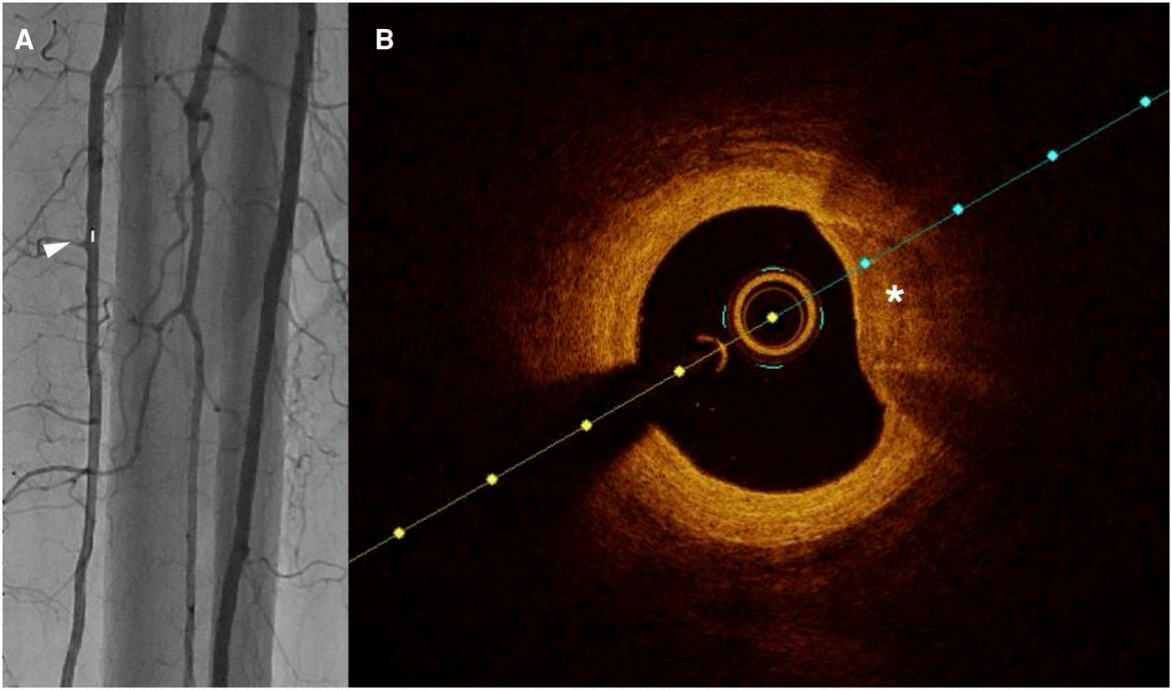
(*A*) Angiography of posterior tibial artery. The white arrowhead on the left panel of A denotes coregistration of the angiographic position to the right panel OCT cross-sectional image (*B*) OCT identified nodular calcification (white asterisk), a heterogeneous area of high and low reflectivity, with low signal attenuation and a sharply demarcated border, protruding to the lumen.

Compared to IVUS, OCT provides 10 times greater image resolution.^52,53^ However, higher resolution comes at the cost of limited field of view and reduced tissue penetration compared to IVUS. Due to reduced penetration depth, OCT may be preferred over IVUS in smaller-calibre blood vessels such as vessels below the knee (*Figure 4*: peroneal artery; *Figure 5*: anterior tibial artery; *Figure 6*: posterior tibial artery). Modern OCT systems employ frequency-domain imaging (FD) technology, which allows for rapid imaging of long vessel segments during brief injections for blood clearance, maintaining good longitudinal resolution.^51,54^

**Figure 7:**
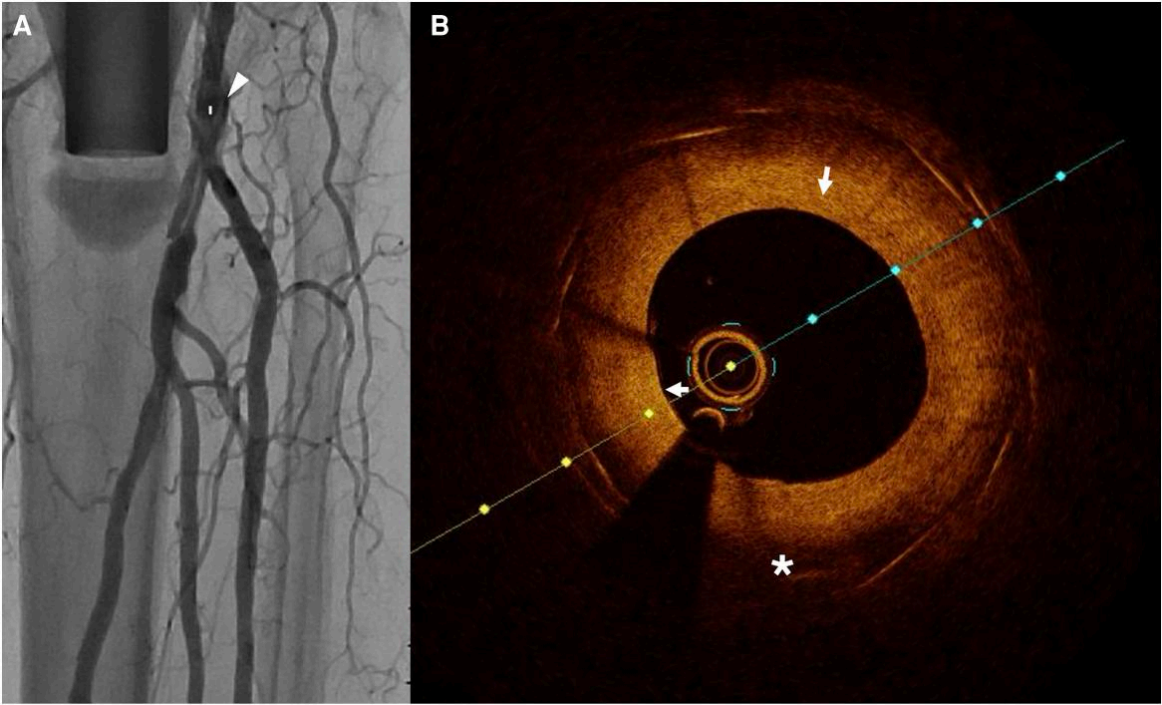
(*A*) Angiography of tibioperoneal trunk. The white arrowhead on the left panel of A denotes coregistration of the angiographic position to the right panel OCT cross-sectional image (*B*) OCT identified instent restenosis with layered neointima shows concentric layers with different tissue properties: adluminal high-intensity (white arrow) and abluminal low-intensity layers (white asterisk). Stent struts behind these tissues were visible.

**Figure 8:**
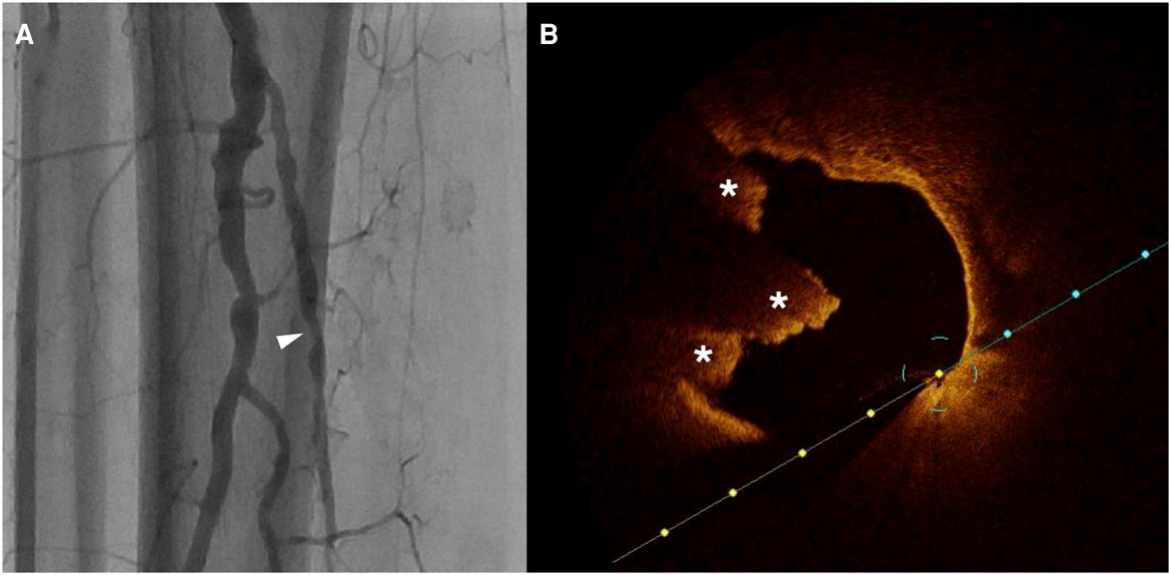
(*A*) Angiography of anterior tibial artery. The white arrowhead on the left panel of A denotes coregistration of the angiographic position to the right panel OCT cross-sectional image (*B*) OCT identified eruptive calcified nodule (white asterisk) with disrupted fibrous cap.

**Figure 9:**
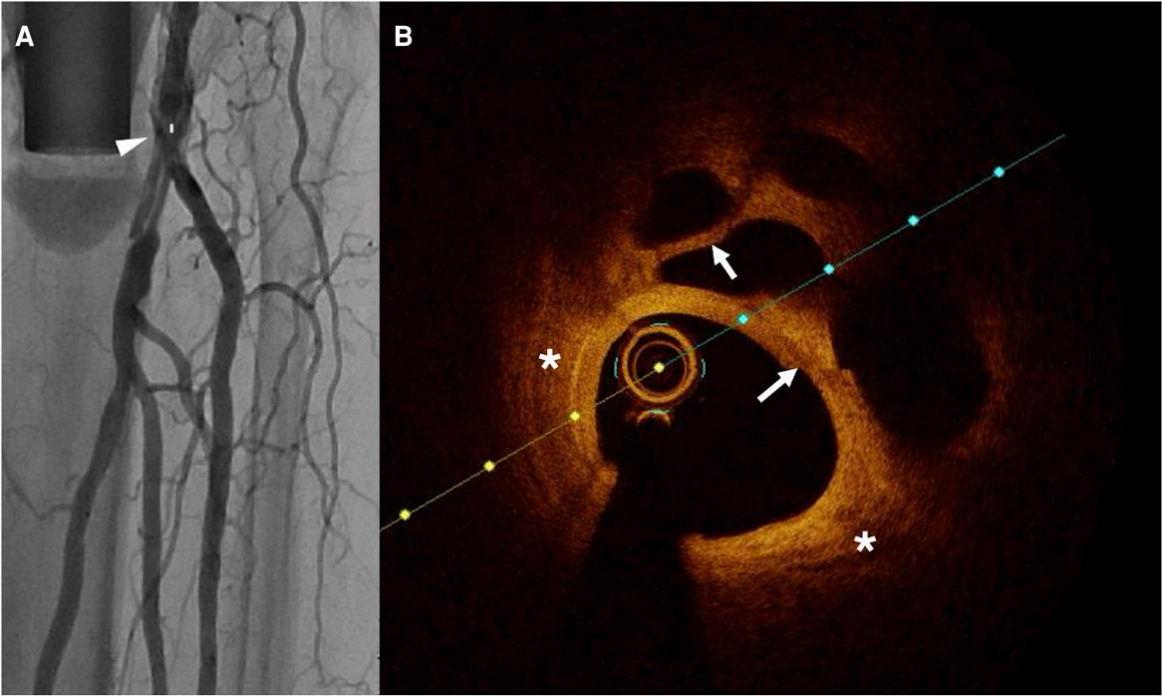
(*A*) Angiography of tibioperoneal trunk. The white arrowhead on the left panel of A denotes coregistration of the angiographic position to the right panel OCT cross-sectional image (*B*) OCT identified ‘Swiss cheese,’ ‘lotus root,’ or ‘honeycomb’ appearance typical of a recanalized thrombus. Heterogeneous plaque with fibrotic tissue (diffuse high-reflectivity intimal thickening with low attenuation, white asterisk). Central lumen divided by thin signal-rich septa (white arrow) into multiple channels.

Compared to IVUS, OCT may provide enhanced visualization of plaques, including eruptive nodules, calcified plaques, stent surfaces, and stent architecture,^52,55^ e.g. when evaluating lesion restenosis and in-stent restenosis (ISR)^52,56–58^. *Figure 7* shows an example of OCT used to evaluate ISR in the tibioperoneal trunk (TPT). *Figure 8* shows an eruptive calcified nodule on OCT. OCT can also characterize post-POBA dissections, providing clear visualization of whether the dissection extends into the media, as well as identifying healed dissections and organized thrombus.^57^  *Figure 9* shows the typical ‘honeycomb’ appearance of recanalized thrombus at the TPT on OCT. In addition, there is some evidence that OCT-guided PEI may reduce fluoroscopy times and lessen radiation exposure.^59^

#### Limitations of OCT

OCT utilization is hampered by the need for contrast administration, which is of particular concern in patients with chronic kidney disease (CKD) due to the nephrotoxic potential of contrast agents and the possibility of inducing renal vasoconstriction.^55,60,61^ This issue is especially relevant in lower extremity interventions, where lesions are often long and multifocal.^60^ Carbon dioxide angiography combined with OCT guidance has been reported to reduce contrast requirements in patients with CKD and PAD.^62^ Additionally, alternative flush media such as dextran or saline can be used instead of iodinated contrast for OCT image acquisition.^60^

To remove blood from the field of view, peripheral OCT may require the use of an over-the-wire balloon catheter that is placed proximal to the lesion of interest. This proximal balloon catheter can induce focal spasm, although it is generally responsive to nitrates. Because of technical problems, such as failure of balloon occlusion or insufficient saline flush, some OCT scans need to be repeated or restarted. Lastly, the large amount of saline flush required for OCT (up to 300 mL) can lead to fluid overload.^55^

Compared to OCT, IVUS provides deeper tissue penetration. The large field of view and larger penetration depth of IVUS make it more suitable in large peripheral arteries, such as lesions above the popliteal artery, compared to OCT.^63^ OCT is limited by a tissue penetration depth of only 1–3 mm, which restricts its application in large peripheral arteries. It may be particularly useful in vessels with a diameter of less than 6 mm.^55,60^ Moreover, on OCT, artefact frequency increases with vessel diameter. Image quality is therefore better for IVUS in the popliteal artery and above.^55^

Reduced tissue penetration comes with higher resolution, which allows OCT to significantly improve tissue discrimination of the vessel wall layers and exhibits better reproducibility in the peripheral arteries.^53^ Image quality of smaller infra-popliteal peripheral arteries, such as the anterior and posterior tibial arteries and the peroneal arteries, is superior on OCT than in IVUS.^55^

If a patient has issues with volume overload, the operator has to consider the saline boluses (of up to 350 mL) that are associated with blood clearance in peripheral OCT imaging.^55^ IVUS does not require blood clearance from the lumen to obtain images. In patients with volume overload, IVUS may be preferable even in a small peripheral vessel.

Additionally, motion artefact (‘Sew-up’), saturation artefact, blooming artefact (caused by stent struts), bubble artefact (from air bubbles), strut-orientation artefact, and fold-over artefacts can also cause image degradation.^51^ The occurrence of fold-over artefacts may be more frequent when OCT is utilized in peripheral arteries, where the vessel diameter exceeds the imaging depth.^51^

## Clinical evidence and outcomes

Overall, there is less evidence for the use of OCT in PEI compared to IVUS. This is partly due to the fact that IVUS has been used for decades (since the 1970s^64^) while OCT was only introduced in the late 1990s.^51^ Although no RCTs support the routine use of OCT for diagnosing and managing PAD, existing literature suggests that OCT is safe and effective. A systematic review by Tung *et al.* identified 15 studies investigating OCT in PAD, including for ISR, fibromuscular dysplasia, and acute limb ischaemia.^59^ Studies consistently reported detailed imaging of vessel characteristics, including plaque composition and intimal tears, before and after interventions.

For the treatment of ISR, OCT gave insights into the mechanism and lesion morphology with its unique ability to visualize neointimal hyperplasia and neo atherosclerosis.^52,65^ Other studies demonstrated OCT's ability to reduce fluoroscopic time compared to angiography guided PEI, and prevent bailout stenting, supporting its viability in both diagnostic and therapeutic scenarios.^59,62,65^ In the setting of CTO, studies showed OCT-guided directional atherectomy procedures to be safe and effective, though limitations such as small sample sizes and exclusion of ISR lesions were noted.^62,66,67^ Selmon *et al.* published a multicentre trial including 100 patients using the Ocelot catheter in CTO crossing (CONNECT II Trial), which met their primary end-points of safety and technical success.^68^ Stavroulakis *et al.* similarly reported in 2019 the successful use of the Pantheris OCT-guided directional atherectomy device in 37 FPA lesions. After successful lesion debulking, all patients underwent adjunctive treatment with DCB, and their primary patency rate was 93% at 12 months. After 18 months, no major amputation was performed in any of the 37 treated patients.^67^

The use of OCT has also been used in studies using atherectomy. Schwindt *et al.* conducted a prospective multicentre trial involving 158 patients and reported the use of the OCT-guided atherectomy Pantheris catheter in treating patients with symptomatic FPA disease (VISION Trial).^66^ OCT-guided directional atherectomy resulted in technical success in almost 100% of lesions treated.^66^

Hoyt *et al*. prospectively studied 13 patients who underwent OCT-guided stenting (Zilver PTX nitinol self-expanding DES with paclitaxel coating) in patients with superficial femoral artery (SFA) disease, using OCT peri-procedurally and at 12-month follow-up to assess vascular patency and inflammation.^69^ The percentage of malposed stent struts decreased from 10.3% postintervention to 1.1% at the 12-month follow-up visit. Additionally, the average expansion of the stent's cross-sectional area was 35% over the entire follow-up period. OCT was crucial in identifying markers of inflammation (macrophage clusters, peri-strut low-intensity area, neovessels) in response to the paclitaxel stent.^69^ This response could potentially lead to stent thrombosis and may explain the elevated mortality that was associated with paclitaxel-coated devices in femoral and popliteal arteries.^69,70^

OCT-guided PI appear to be comprehensively effective when their use is paired with DCB.^67^ DCBs deliver antiproliferative drugs during balloon inflation without the need for a permanent metallic implant, which may be favourable in PAD, where the mechanical stress caused by the metal may lead to neointimal hyperplasia.^48^ A recent meta-analysis comparing treatment modalities in the lower extremities (POBA, DES, BMS, and DCB) showed that DCB had the lowest need for target lesion revascularization post intervention.^71^  *Tables 8* and *9* summarize available studies that have evaluated the use of OCT in peripheral interventions.

**Table 8:** Available studies examining OCT Use in peripheral interventions (2011–2014)

Study	Number of patients	Lesion details	Study population	Follow up	Findings	Limitations
Karnabatidis *et al.* (2011)	20 patients with 27 lesions	Lesions include *de novo* atherosclerotic lesions (*n* = 18) and in-stent restenosis (ISR) (*n* = 9). Lesion complexity by TASC: IIA (*n* = 7), IIB (*n* = 17), IIC (*n* = 3), IID (*n* = 0). CTOs present (*n* = 3). Mean lesion length 5.3 ± 2.2 cm. Calcification present (*n* = 5). OCT plaque morphology in *de novo* lesions: fibrotic (*n* = 5), fibrocalcific (*n* = 8), lipid-rich (*n* = 3), necrotic/calcified (*n* = 2); ISR lesions uniformly fibrotic (*n* = 9).	Patients with femoropopliteal disease undergoing **POBA** and imaging with frequency domain OCT	n/a	High resolution imaging obtained in 93.9% of pullbacks and was able to further characterize plaque and ISR lesions OCT was able to identify intimal tears and dissections after angioplasty that angiography failed to identify and would sometimes lead to stenting Additional procedural time of 50 min attributed to OCT No OCT related complications	Small sample size Lack of follow up and repeat imaging
Eberhardt *et al.* (2013)	16 patients (112 arterial segments imaged)	Short femoropopliteal lesions ≤10 cm (TASC II A only). No TASC B–D lesions or CTOs included. Atherosclerotic plaques present in all analysed segments; mean OCT/IVUS scan length 3.0 cm per segment.	Patients with symptomatic peripheral artery occlusive disease	n/a	OCT outperformed IVUS regarding image quality, plaque assessment and vessel wall layer discrimination Interreader reproducibility with OCT was higher compared to IVUS OCT use led to higher cumulative fluoroscopy time and radiation dose compared to IVUS	Small sample size
Paraskevopoulos (2013)	12 patients; 19 stents analysed	Infrapopliteal DES lesions Target vessels: anterior tibial (*n* = 10), tibioperoneal trunk (*n* = 4), posterior tibial (*n* = 3), peroneal artery (*n* = 2). Binary ISR present (*n* = 10). OCT-defined neointimal features: lipid-laden neointima (*n* = 16), neointimal calcification (*n* = 6), neovascularization (*n* = 13), thrombus (*n* = 5), neointimal rupture (*n* = 4).	Prospective study describing use of FD-OCT to characterize ISR in patients with previous infra-popliteal stents	13 months	Percent restenosis was higher after longer follow-up and in symptomatic vs. asymptomatic patients	
Brodmann *et al.* (2013)	10 patients	n/a	Patients with previous SFA POBA or stenting with restenosis	n/a	Patients with previous POBA had classic atherosclerotic plaques with mixed lipidic, calcific, and fibrotic areas. ISR lesions had massive neo-intimal hyperplasia	Observational study
Selmon *et al.* (2013) CONNECT II	100 patients	SFA (*n* = 94), popliteal artery (*n* = 4), combined femoropopliteal segment (*n* = 2). Mean lesion length 16.6 ± 9.3 cm. Lesion type: *de novo* (*n* = 89), restenosis (*n* = 11). Calcification severity by angiography: none (*n* = 11), mild (*n* = 53), moderate (*n* = 36); severely calcified lesions excluded by protocol.	Patients with femoropopliteal CTO treated with Ocelot catheter	30 days	The Ocelot catheter successfully cross 975 of target CTOs	
De Donato *et al.* (2014)	15 (58 OCT pullbacks)	n/a	Patients with femoropopliteal disease undergoing endovascular treatment	n/a	Technical success of OCT pullback was 94.8%, suggesting feasibility OCT demonstrated good inter and intraobserver agreement OCT was able to demonstrate intimal tears, flaps, residual dissection, etc No OCT related complications	Small sample size
Yamane *et al.* (2014)	330 patients with 365 ISR lesions	OCT tissue morphology classified as homogeneous (*n* = 149) and non-homogeneous (*n* = 216).	Patients with ISR treated with POBA vs. DCB vs. stenting based on OCT lesion appearance	1 year	In patients whose lesions by OCT appeared non-homogenous, recurrent TLR occurred in 33% of POBA group, in 14% of PCB group and in 13% of SI group (*P* = 0.002).	

Available studies examining OCT use in peripheral interventions (2011–2014).

CTO, chronic total occlusion; DCB, drug-coated balloon; FD-OCT, frequency-domain optical coherence tomography; ISR, in-stent restenosis; IVUS, intravascular ultrasound; OCT, optical coherence tomography; POBA, plain old balloon angioplasty; SFA, superficial femoral artery; TASC II, TransAtlantic Inter-Society Consensus II; TLR, target lesion revascularization.

**Table 9:** Available studies examining OCT use in peripheral interventions (2015–2020)

Study	Number of patients	Lesion details	Study population	Follow up	Findings	Limitations
Micari *et al*. (2015)	45 patients	PA interventions *n* = 18, infrapopliteal interventions *n* = 17, combined FPA and BTK interventions *n* = 10.	Patients with PAD of the SFA undergoing endovascular intervention	n/a	Demonstrated manual injection of 50 mL of pure saline instead of contrast during OCT imaging is able to achieve detection of vessel plaque and dissections	Not directly compared to contrast OCT group
Schwindt *et al*. (2017), prospective, single-arm (VISION)	158 patients with 198 lesions	Symptomatic FPA lesions treated with OCT-guided directional atherectomy (Pantheris); patients *n* = 158, lesions *n* = 198. Lesion location: SFA (*n* = 160), SFA + popliteal (*n* = 12), popliteal artery (*n* = 26). Lesion type: stenosis (*n* = 158) and CTO (*n* = 40). Lesion complexity by TASC II: A (*n* = 150), B (*n* = 40), C (*n* = 6); TASC D excluded. Mean lesion length 5.3 ± 4.0 cm by angiography and 7.2 ± 3.6 cm by OCT-guided treatment length. Calcification severity by angiography: none (*n* = 43), mild (*n* = 153), moderate (*n* = 2); severely calcified lesions excluded. De novo lesions only; ISR excluded.	Patients undergoing OCT guided atherectomy catheter for symptomatic femoropopliteal artery disease	30 days, 6 months	OCT guided atherectomy results in technical success in 97% of lesions treated OCT guided atherectomy was effective at reducing mean diameter stenosis	Small sample size Short follow up No true control group More complex lesions (restenosis, heavily calcified, acute lesions, etc.)
Holden *et al*. (2019)	25 patients (10 OCT images)	Lesion location: SFA (*n* = 20), popliteal artery (*n* = 5). Lesion type: *de novo* (*n* = 24) and native restenosis (*n* = 1). CTOs ≤6 cm present (*n* = 8). Mean lesion length 4.7 cm (range 1.5–9.8 cm). Calcification by angiography: moderate (*n* = 7) and severe (*n* = 7); severely circumferential calcification excluded by protocol.	Patients with femoropopliteal disease undergoing intervention with serration *POBA*	n/a	OCT was able to identify and confirm serrations of lesions following treatment	Only 6 of patients underwent OCT confirmation of serrations Single arm study
Stavroulakis *et al*. (2019), retrospective, single-arm	33 patients	Lesion type: *de novo* atherosclerotic lesions (*n* = 25) and restenosis (*n* = 12). CTOs present (*n* = 13). Lesion location: distal SFA (*n* = 20), mid SFA (*n* = 12), proximal popliteal artery (*n* = 8). Mean lesion length 7.0 cm (IQR 4.0–10.4). Calcification severity by angiography: mild (*n* = 2), moderate (*n* = 5), severe (*n* = 1).	Patients undergoing OCT guided atherectomy catheter and *DCB* for symptomatic FPA lesions	15 months	Combination of OCT guided atherectomy with drug coated balloon angioplasty is safe and effective Primary patency rate was 93% at 12 months and 78% at 18 months	Small sample size Retrospective Limited number of severely calcified lesions
Hoyt *et al*. (2020), prospective, single-arm	13 patients	Target lesions included significant SFA stenosis (≥60%) and CTOs (100% occlusion). Mean target lesion length 18.5 ± 13.2 cm.	Symptomatic superficial femoral artery disease (total occlusion or stenosis > 60%) undergoing *DES*	12 months	Demonstrated OCTs ability to characterize measures of inflammation (neovascularization, macrophage arcs, thrombus) OCT also demonstrated minimal neointimal growth and outward remodelling after paclitaxel coated stent implantation	Small sample size High rate of patient drop out Lack of control group
Memon *et al.* (2021)	11 patients, 18 vessels	n/a	Patients with baseline CKD IV and PAD and femoropopliteal CTOs treated with Ocelot catheter	1 year	Carbon dioxide angiography and OCT guided CTO crossing/directional atherectomy resulted in decreased use of contrast agents and radiation exposure	

Available studies examining OCT use in peripheral interventions (2015–2020).

CKD, chronic kidney disease; CTO, chronic total occlusion; DCB, drug-coated balloon; DES, drug-eluting stent; OCT, optical coherence tomography; PAD, peripheral artery disease; POBA, plain old balloon angioplasty; SFA, superficial femoral artery.

**Figure 10:**
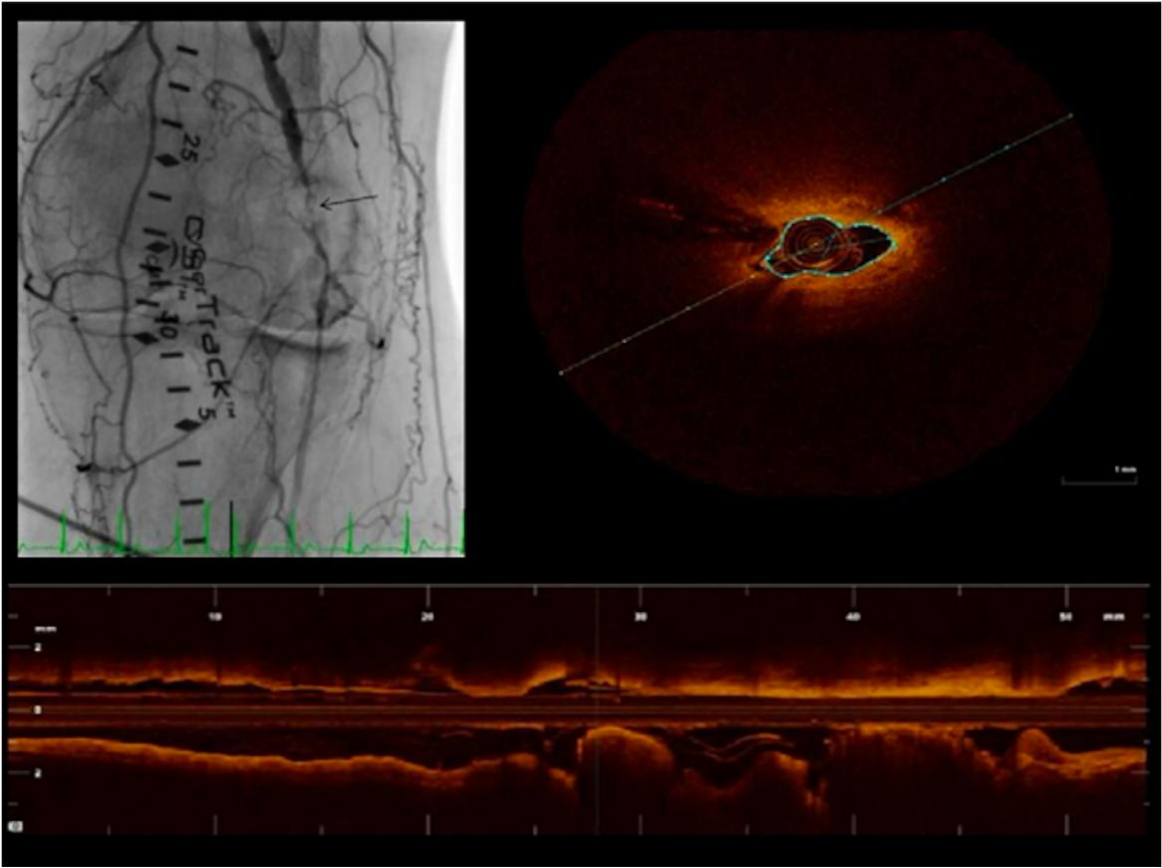
Sub-total occlusion of the distal popliteal artery extending to the tibioperoneal trunk (arrow, left image) showing heavy calcification. Reproduced with permission from K. Marmagkiolis *et al.* Optical Coherence Tomography to guide Below-the-Knee Endovascular Interventions, International Journal of Cardiology, 2014.

In infra-popliteal vessels, which are generally smaller in diameter compared to supra-popliteal vessels, OCT may be particularly useful. Paraskevopoulos *et al*. demonstrated the utility of OCT in evaluating the mechanism of ISR in infra-popliteal DES.^58^ OCT identified neoatherosclerosis, neovascularization, and neointimal rupture.^58^ Marmagkiolis *et al.* reported a case of an OCT-guided PEI of a distal popliteal to the TPT sub-totally occluded lesion.^72^ OCT demonstrated significant nearly circumferential calcification (*Figure 10*), necessitating lesion preparation with an orbital atherectomy device.^72^ These studies underscored the safety and feasibility of OCT in infra-popliteal vessels.

## When to choose IVUS or OCT in peripheral interventions

As discussed, both IVUS and OCT provide significant advantages over DSA. However, significant notable differences between the two must be considered. Superficial calcium deposits in the vessel wall can reduce the penetration of sound waves, which impacts the image quality in IVUS.^73^ OCT detects calcium as signal-poor areas with well-delineated sharp borders, allowing for precise evaluation of calcium thickness.^74,75^ However, due to less penetration depth, OCT may be less favourable to visualize deep calcium, especially in large above the knee vessels. Lastly, OCT images have faster imaging acquisition than IVUS.^53,76^

Despite their significant differences, quantitative image analysis with OCT and IVUS demonstrates high correlation. Mean luminal area, vessel area, and distal, middle, and proximal lumen diameters have been shown to be similar between both modalities.^53,55^ Although OCT allows visualization in great detail to identify plaque, calcium deposition, and indwelling devices such as vascular stents, IVUS provides sufficiently clear imaging to allow for a diagnostic and treatment strategy.^53^

In the setting of ISR, OCT allows for markedly increased resolution and image quality, less limitation by stent struts, and can be used to visualize neointimal hyperplasia.^55^ If there is a need for atherectomy, there are multiple commercially available OCT-guided atherectomy catheters, which excise tissue with minimal injury to healthy tissue (*Table 7*).

## Clinical implications of lesion heterogeneity

Lesion type and plaque morphology differed meaningfully across studies and had direct implications for procedural strategy and outcomes. In IVUS-guided trials enrolling FPA lesions, imaging was most frequently applied in anatomically and biologically complex disease. Allan *et al.* demonstrated that a substantial proportion of treated lesions exhibited severe calcification (PACSS 3–4), a phenotype known to impair stent expansion, limit drug uptake, and increase restenosis risk.^34^ Similarly, Kurata *et al.* and Krishnan *et al.* showed that IVUS guidance was commonly used in long lesions exceeding 13–20 cm, with high rates of CTO and advanced TASC C–D disease, lesion subsets in which angiography alone underestimates vessel size and calcium burden, leading to suboptimal device sizing and incomplete lesion preparation.^13,77^ In these settings, intravascular imaging enabled more accurate assessment of reference vessel diameter, calcium distribution, and lesion length, factors that are mechanistically linked to improved acute luminal gain and reduced target lesion failure.

OCT-based studies further illustrated how plaque microstructure influences procedural risk and postintervention healing. Karnabatidis *et al.* and Paraskevopoulos *et al.* demonstrated that OCT could differentiate fibrotic, fibrocalcific, lipid-rich, and thrombotic plaque components, while identifying neointimal calcification, thrombus, and heterogeneous tissue patterns in ISR.^58,78^ These features are clinically relevant, as lipid-rich or thrombotic lesions are associated with distal embolization and acute recoil, whereas calcified and fibrocalcific plaques predict higher restenosis rates.^79,80^ In long FPA CTOs, Selmon *et al.* and Schwindt *et al.* showed that OCT guidance facilitated true lumen crossing, findings that directly inform the need for additional vessel preparation or stent coverage.^66,68^ Serial OCT studies by Hoyt *et al.* further linked plaque morphology to vascular healing, demonstrating associations between neointimal heterogeneity, malapposition, and late luminal loss following DES implantation.^69^

Taken together, the available evidence indicates that lesion characteristics and plaque morphology are not merely descriptive variables but key modifiers of both procedural decision-making and clinical outcomes.

## Technological advancements and future directions

There are currently 10 commercially available peripheral IVUS catheters from 4 companies (Philips, Boston Scientific, ACIST, InfraRedex) and 7 peripheral OCT catheters from Avinger and Gentuity (*Tables 1* and *7*). Recent innovations in high definition (HD) IVUS are overcoming the many drawbacks of conventional IVUS, including allowing for higher spatial resolution and faster image acquisition.^53^ In the OCT realm, a first-of-its-kind randomized, controlled, and multicentric trial Optical Coherence Tomography Contribution Assessment in the Revascularization of Long Femoropopliteal Occlusion Lesions (TASC C and D): A Randomized Trial will assess the benefit of intraoperative OCT in addition to angiography in patients with long *de novo* FPA lesions. Their primary outcome will be primary patency at 12 months, and secondary endpoints will include symptomatic improvement, TLR, TVR, quality of life questionnaires, cost utility, and cost effectiveness at 12 months.^81^ Lastly, there have been advances in the development of combined IVUS-OCT catheters. There are currently two available combined IVUS-OCT catheters from CONAVI and TERUMO.^82^ Their crossing profile (Imaging Window: 2.8F for CONAVI; 2.5F for TERUMO) is suitable for the coronary realm, and it may be favourable in larger peripheral vessels, while also addressing the weaknesses of each individual imaging modality.^83,84^

A Chinese company, PANOVISION, conducted a first-in-human large scale RCT using a hybrid IVUS-OCT catheter in 99 patients. Compared to a stand-alone IVUS or OCT, the IVUS or OCT of the hybrid imaging system was significantly non-inferior regarding the primary endpoint (clear stent capture rate), the dual-modality images were successfully acquired in all 99 participants, and the detection rate of tissue prolapse was significantly higher by hybrid IVUS compared to stand-alone IVUS.^85^ The same PANOVISION hybrid catheter was used to develop an optical ultrasonic flow ratio (OUFR), which in a large prospective study, was found to have excellent agreement with invasive fractional flow reserve (FFR), and was superior to single imaging modality-based physiology indexes.^86^ Emerging technologies, such as hybrid intravascular imaging catheters like TERUMO, CONAVI, and PANOVISION, are leading the way for future research. An ‘all-in-one’ imaging modality, which combines the strengths and overcomes the relative weaknesses of OCT and IVUS, has great potential for PEI optimization.

## Conclusion

Intravascular imaging (IVUS or OCT) for PEI provides valuable information about lesion characteristics that guides operator and reduces procedure related complications. This information is critical, as often lesions are treated without stent deployment that would normally provide vessel support or seal off procedure related dissections.

In comparison to DSA, both IVUS and OCT can provide superior assessment of lesion severity, plaque composition, vessel dimensions and stent expansion and apposition. However, operators should be familiar with the strengths and weaknesses of each imaging modality, so they can properly select the optimal technology for their specific use case. A deep understanding of these nuances can help operators achieve superior clinical outcomes.

The use of either IVUS or OCT has been identified in retrospective and prospective studies to improve patient outcomes. The goal of this review was to describe the totality of available data on IVUS and OCT use in peripheral interventions, so that on case-by-case basis, either IVUS or OCT is utilized to improve patient outcomes.

## Supplementary Material

oeag016_Supplementary_Data

## Data Availability

This study is a scoping review of previously published literature. All data supporting the findings of this study are available within the article and its Supplementary material.
