# Correction to: Intravascular imaging in peripheral arterial disease: a contemporary literature review

**DOI:** 10.1093/ehjopen/oeag117

**Published:** 2026-07-17

This is a correction to: Jason Galo, Abdullah Al-Qaraghuli, Ryan Wallace, Aninka Saboe, Julianna Morera, Pablo Rubio, Abhishek Chaturvedi, Beni Verma, Kalyan R Chitturi, Ron Waksman, Hector M Garcia-Garcia, Intravascular imaging in peripheral arterial disease: a contemporary literature review, *European Heart Journal Open*, Volume 6, Issue 2, March 2026, oeag016, https://doi.org/10.1093/ehjopen/oeag016

The following change has been made to the originally published paper.

Under sub heading **When to choose IVUS or OCT in peripheral interventions,** the last sentence of the 2nd paragraph, has been deleted: “The Central Illustration summarizes an algorithmic approach of when to utilize IVUS or OCT in PEI.”

