# Supplementary material for: Intravascular imaging in peripheral arterial disease: a contemporary literature review
Source: Eur Heart J Open. 2026 Feb 13;6(2):oeag016. doi: 10.1093/ehjopen/oeag016 (PMC12967067; doi:10.1093/ehjopen/oeag016)
Supplement: oeag016_Supplementary_Data [file oeag016_supplementary_data.docx]

**Supplement**

1. **Methods (continued)**

Following title and abstract screening, 158 reports were identified as potentially relevant and were sought for full text retrieval. All reports were successfully retrieved, leaving 158 full text articles assessed for eligibility. Studies were eligible for inclusion if they involved adult patients undergoing peripheral endovascular interventions for lower extremity PAD, evaluated the use of IVUS or OCT during the procedure either diagnostically or as an adjunct to intervention, reported clinical, procedural, or imaging-guided outcomes, and were published as full text articles in English. All study designs were considered, including retrospective analyses, prospective observational studies, randomized controlled trials, and systematic reviews or meta-analyses.

Studies were excluded if they focused exclusively on coronary, carotid, or non-lower-extremity vascular territories; were case reports, editorials, expert opinions, conference abstracts without full data, or animal studies; or did not report clinical or procedural outcomes related to intravascular imaging use. Consistent with scoping review methodology, the study selection process was designed to broadly capture literature relevant to the scope of the review, rather than to apply the restrictive eligibility criteria. Ultimately, 42 studies met all inclusion criteria and were included in the review.

Data extraction was performed independently by two reviewers and was descriptive in nature. Extracted variables, as reflected in the tables, included study design, sample size, patient population, vascular territory, imaging modality, procedural context, follow-up duration, and principal findings.

Studies were grouped by imaging modality, study design, and publication era. Findings were summarized as reported by the original studies without pooled analyses. The tables served as the primary framework for organizing the evidence and guided the narrative synthesis, allowing comparison across study types and identification of consistent findings and gaps in the literature. Study limitations and methodological considerations were qualitatively assessed and discussed where relevant.
